# Supplementary material for: The deubiquitinase Usp9x regulates PRC2-mediated chromatin reprogramming during mouse development
Source: Nat Commun. 2021 Mar 25;12:1865. doi: 10.1038/s41467-021-21910-0 (PMC7994559; doi:10.1038/s41467-021-21910-0)
Supplement: Supplementary file 1 — Supplementary Information [file 41467_2021_21910_MOESM1_ESM.pdf]

SUPPLEMENTARY INFORMATION

The deubiquitinase Usp9x regulates PRC2-mediated chromatin reprogramming during mouse development

Trisha A. Macrae[1,2,3,5], Miguel Ramalho-Santos[1,2,4,5*]

Author information:
[1]Eli and Edythe Broad Center of Regeneration Medicine and Stem Cell Research, University of California, San Francisco, San Francisco, CA, USA.
[2]Center for Reproductive Sciences, University of California, San Francisco, San Francisco, CA, USA.
[3]Medical Scientist Training Program, University of California, San Francisco, San Francisco, CA, USA.
[4]Department of Molecular Genetics, University of Toronto, Toronto, Ontario, Canada.
[5]Lunenfeld-Tanenbaum Research Institute, Mount Sinai Hospital, Toronto, Ontario, Canada.

*email: mrsantos@lunenfeld.ca.

Supplementary information includes figures and references.

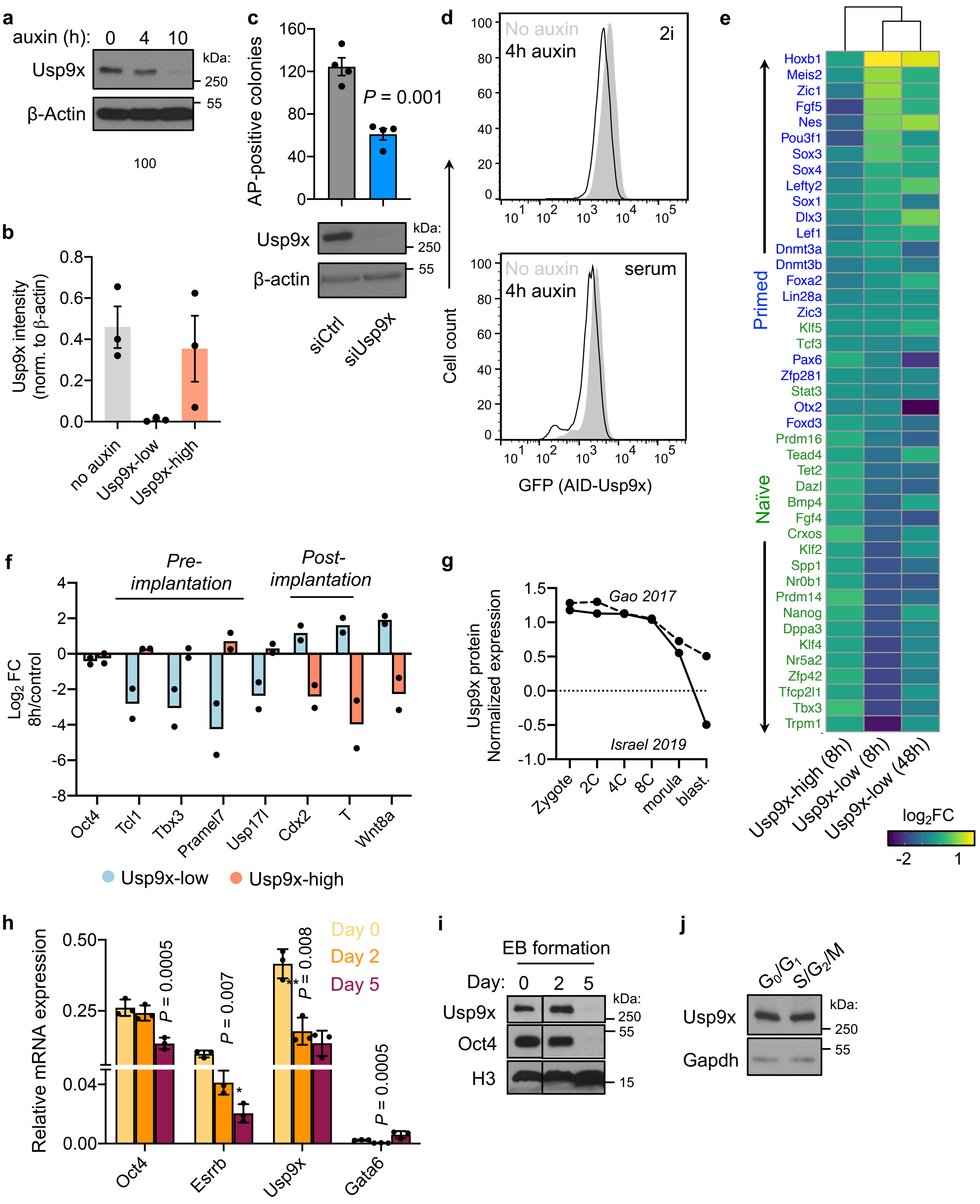

Supplementary Figure 1. Characterization of Usp9x expression in targeted ES cells and early embryos. a) Auxin treatment induces acute depletion of endogenous Usp9x protein over a time course of auxin, 0-10h. **b)** Quantification of indicated samples from western blots of cells sorted after auxin treatment (see Fig. 1a). **c)** Colony formation assay in control (siCtrl) or Usp9x-

depleted (siUsp9x) ES cells with western blot confirming Usp9x knockdown. *AP*, Alkaline Phosphatase. **d)** Flow cytometry plots comparing Usp9x expression and response to 4h auxin depletion in 2i versus serum conditions. **e)** Relative expression of representative naïve and primed pluripotency genes in the indicated cell states[1,2]. Data are $\log_2$ fold-change (FC) in expression relative to controls. **f)** qRT-PCR validation of representative genes from RNA-seq at 8h after auxin. *FC*, fold-change. **g)** Usp9x protein expression declines over pre-implantation development, in parallel with the decline in Usp9x mRNA in early development (Fig. 1e). Normalized data are plotted from quantitative proteomic analyses of wild-type embryos[3,4]. **h)** qRT-PCR of Usp9x and sample genes during lineage commitment of ES cells in Embryoid Body (EB) formation. Expression normalized to average of H2A, Ubb and Rpl7. *NS*, not significant. **i)** Usp9x protein expression declines during the initial stages of lineage commitment. **j)** Usp9x expression is comparable between stages of the cell cycle, isolated using a FUCCI live cell cycle reporter[5].

Data are representative of 2-3 independent experiments (a,b,d,i,j), mean ± s.e.m. of 3 biological replicates (b, top), mean ± s.e.m. of 4 replicates (representative of 3 independent experiments) (c), mean of 3 (e) or 2 (f,g) biological replicates, mean ± s.d. of 3 biological replicates (h). *P* values by two-tailed Student's t-test with Welch's correction (c), two-tailed t-tests with Holm-Sidak multiple corrections (h).

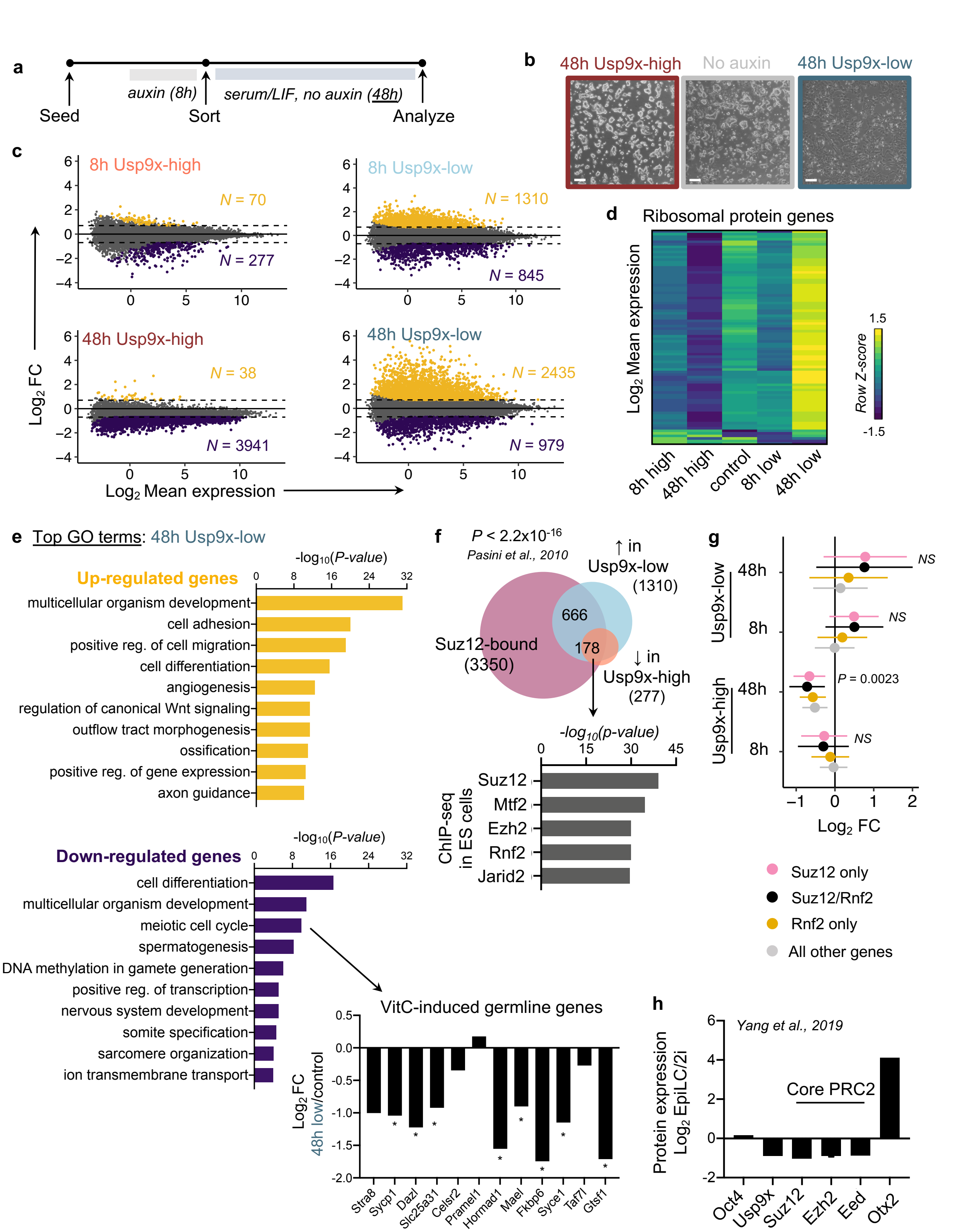

Supplementary Figure 2. Transcriptional analysis of Usp9x-high and Usp9x-low ES cells at 48h. a) Diagram of experiments assessing the ability of sorted Usp9x-high or Usp9x-low ES cells to recover after acute auxin treatment. **b)** After recovery, 48h Usp9x-high ES cells form compact

colonies and Usp9x-low ES cells adopt heterogeneous, differentiated morphologies. Scale bar = 100 µm. **c)** MA plots of expression changes in Usp9x-high and Usp9x-low ES cells relative to no-auxin controls. *N;* number of differentially expressed genes (adjusted *P* < 0.05 and 1.5x fold change). **d)** Relative expression of ribosomal protein genes in Usp9x-high, Usp9x-low, or control cells at 48h. **e)** Gene Ontology (GO) analysis of genes significantly dysregulated in Usp9x-low ES cells after 48h. Inset: fold-change in expression (from DESeq2) of several vitamin C-induced germline genes[6,7]. **f)** ChEA[8] analysis of genes DE in Usp9x-high and Usp9x-low ES cells, with top-enriched factors and overlap of Suz12-bound genes[9] shown. **g)** Log_2 fold-change in expression of the indicated genes subsets in Usp9x-associated cell states, with pairwise comparisons of Suz12-bound versus Suz12/Rnf2-bound gene expression[10]. **h)** Expression of the indicated proteins by quantitative analysis of naïve (2i) ES cells versus primed epiblast-like cells (EpiLCs)[11].

Data are representative of at least 4 experiments (b), log_2FC of 3 biological replicates (c-f), mean ± s.d. of 3 biological replicates (g), mean of 4 biological replicates (h). **FDR* < 0.05 or *P* value as indicated. *P*-values by Fisher exact test (e,f), Wald test with multiple comparison testing by DESeq2 (c, e inset), two-tailed *t*-tests with FDR correction (g).

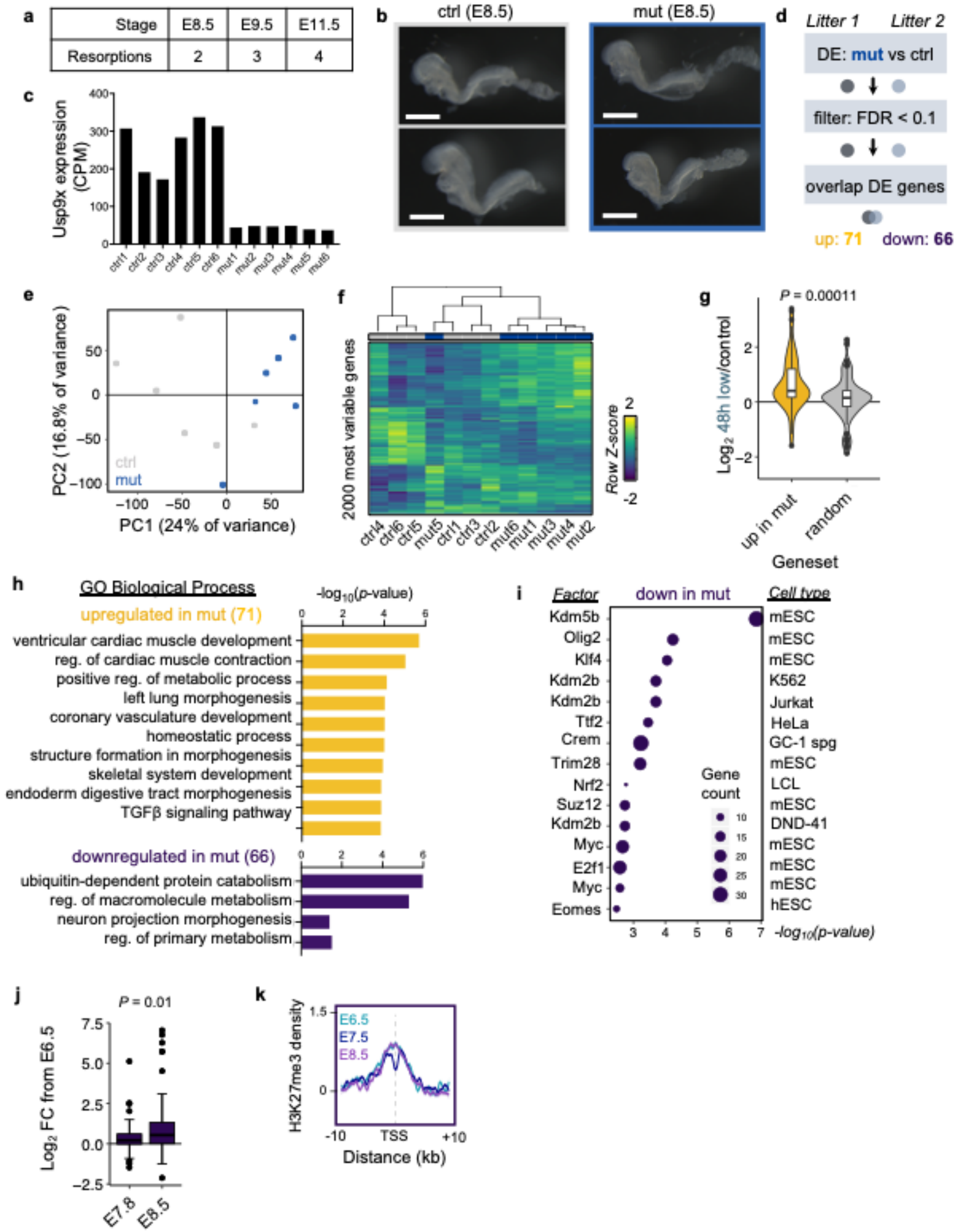

Supplementary Figure 3. Transcriptional analyses of *Usp9x*-mutant embryos at E8.5.

a) Number of resorptions counted at the indicated stages (no embryonic material detected in deciduum). **b)** Sample control and mutant embryos at E8.5 used for RNA-seq (representing *N* =

31 control, *N* = 23 mutant, see Fig. 2a). Mutants are morphologically indistinguishable from controls at this stage. Scale bar = 500 µm. **c)** Normalized counts confirming low Usp9x mRNA expression in the 6 mutant embryos used for RNA-seq. *N* = 12 embryos from 2 litters were sequenced. *CPM*, counts per million. **d)** Approach to differential expression (DE) analysis of E8.5 *Usp9x*-mutant transcriptomes. *FDR*, false discovery rate. **e)** Principle Component (PC) Analysis plots of RNA-seq from litter-matched mutants and controls, showing separation of genotypes along PC1. **f)** Unsupervised hierarchical clustering of the top 2000 most variable genes across samples by RNA-seq. Mutants largely cluster away from controls, except for mut5 (litter 2), which clusters with the controls from litter 1. **g)** Boxplot showing that the genes upregulated in *Usp9x* mutants are also up in 48h Usp9x-low ES cells relative to controls, compared to a random subset of the same number of genes (71). **h)** Top-enriched GO terms for up- and down-regulated genes in *Usp9x* mutants. **i)** Enrichr TF analysis of genes downregulated in *Usp9x*-mutants, similar to Fig. 2e. These genes are targets of repressive chromatin factors, e.g. Kdm5b, Kdm2b, Trim28, and Suz12, in the indicated cell types. **j)** The genes downregulated in *Usp9x* mutants tend to be upregulated by E8.5[12]. **k)** Profile of H3K27me3 ChIP-seq signal during wild-type development over the genes downregulated in *Usp9x* mutants[13].

Boxplot hinges show the first and third quartiles, whiskers show ±1.5*IQR and center line shows median of 3 (g) or 2-3 (j) biological replicates. Data are mean ± s.e.m. of 2-3 replicates per time point (k). *P* values by two-tailed Wilcoxon rank-sum tests (g, j) and Fisher exact test (h,i).

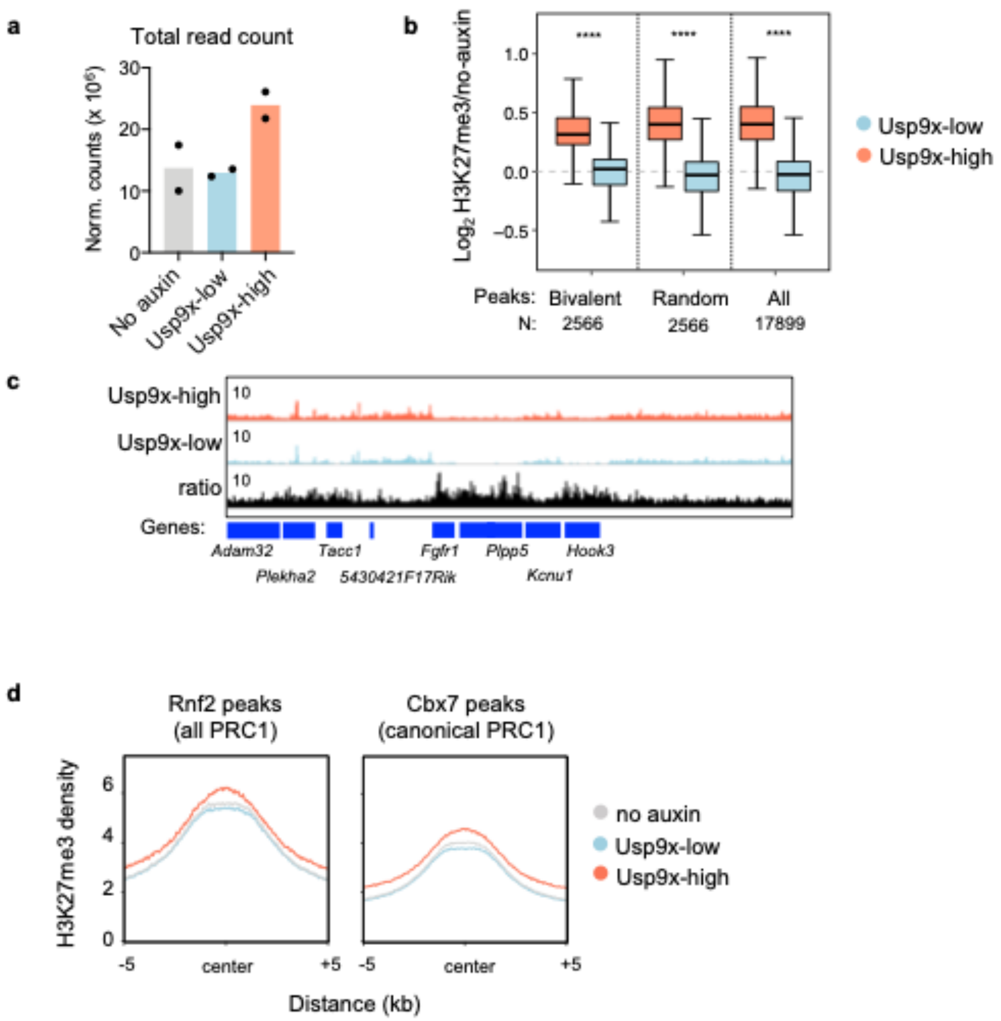

Supplementary Figure 4. Analysis of H3K27me3 patterns in Usp9x-high and Usp9x-low ES cells. a) Spike in-normalized sequencing coverage of H3K27me3 in the indicated cells. **b)** H3K27me3 coverage in Usp9x-high or Usp9x-low cells over bivalent peaks[14], a random peak set of the same size (N = 2566), or all peaks found in ES cells at baseline (no auxin). **c)** Representative genome browser view of H3K27me3 signal in Usp9x-high and Usp9x-low cells. Elevated H3K27me3 signal in Usp9x-high cells is often observed outside of promoters. **d)** Profile plots of H3K27me3 over subsets of PRC1 peaks (± 5kb)[15].

Data are mean ± s.d. (a) or sum (b-d) of 2 biological replicates. Boxplot hinges (b) show the first and third quartiles, with median center line of 2 biological replicates. ****$P < 2.2 \times 10^{-16}$ by two-tailed Wilcoxon rank-sum tests (c).

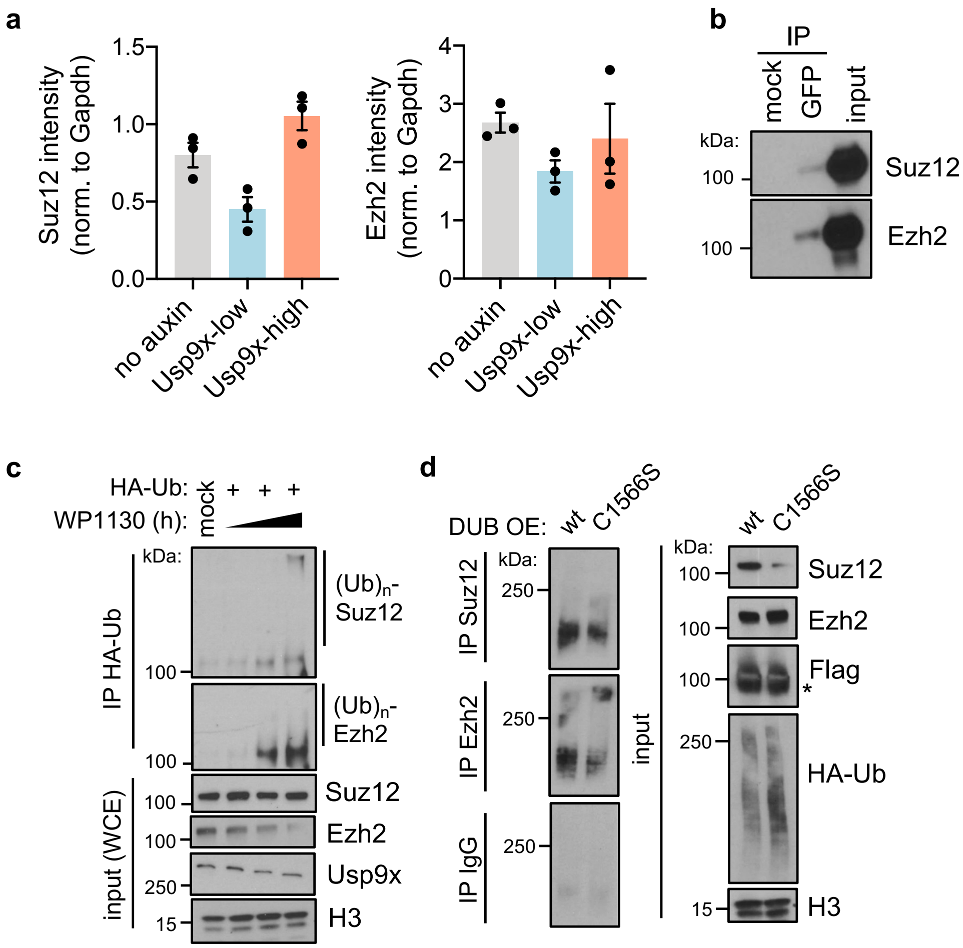

Supplementary Figure 5. Validation of the Usp9x-PRC2 regulatory interaction. a) Quantification of Suz12 and Ezh2 in no-auxin, Usp9x-low and Usp9x-high cell fractions (see Fig. 4a). **b)** Co-IP showing that GFP-tagged Usp9x interacts with endogenous Suz12 and Ezh2 in AID-Usp9x cells. **c)** Acute catalytic inhibition of Usp9x with the semi-selective inhibitor WP1130 leads to gain of ubiquitin at PRC2 proteins, similar to Fig. 4c. WP1130 treatment ranges from 0-4h. **d)** Comparison of wt versus catalytic-dead Usp9x catalytic domain overexpression but in wild-type ES cells, similar to Fig. 4d. Asterisk (*) designates the expected band size for the Usp9x catalytic domain construct. Error bars show mean ± s.e.m. (a). Western blots are representative of 2-3 biological replicates.

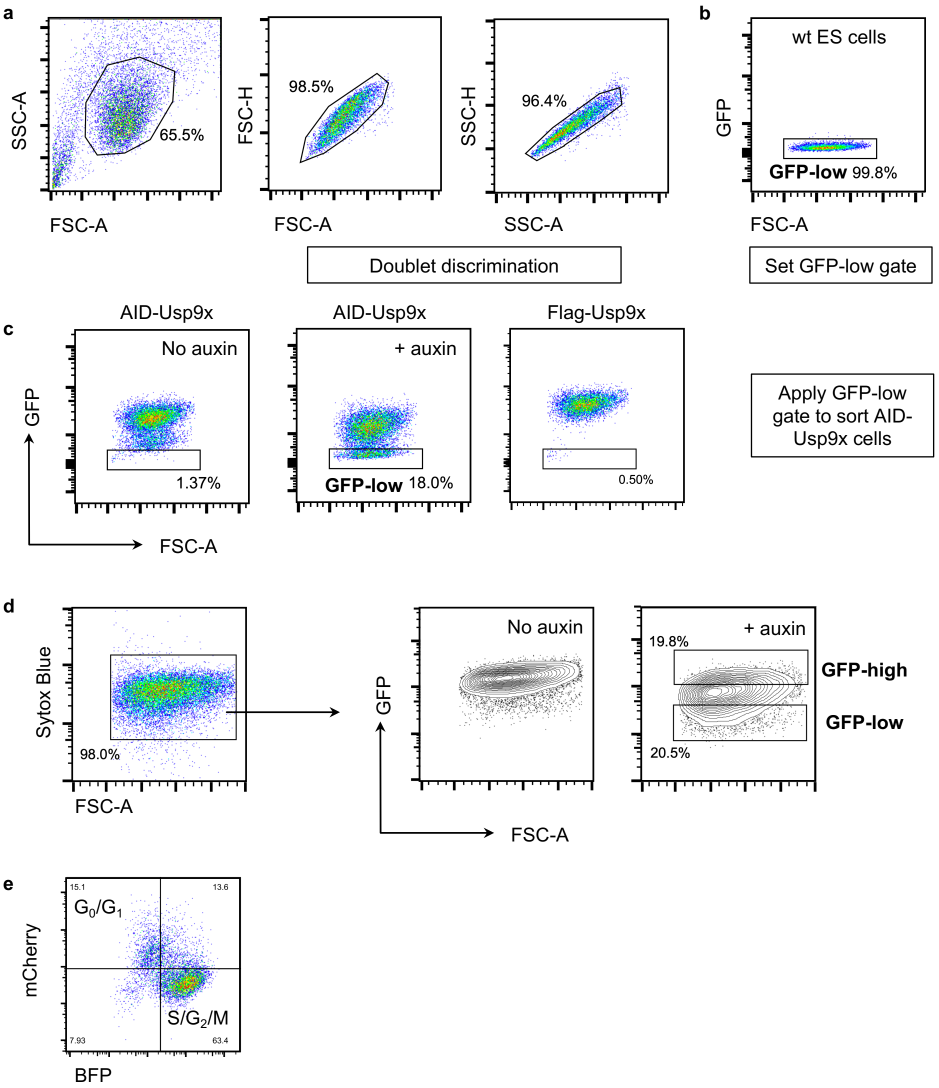

Supplementary Figure 6. Gating strategy for flow cytometry and FACS experiments. a) Fluorescence-activated cell sorting (FACS) demonstrating isolation of single cells. **b)** Wild-type ES cells were used to define negative GFP expression. **c)** Application of the GFP-low gate defined in (b) to AID-Usp9x ES cells with or without auxin. 15-20% of auxin-treated cells fall into the GFP-low gate. No-auxin and Flag-Usp9x ES cells have a negligible GFP-low population. **d)** For subsequent experiments, Sytox Blue incorporation was used to exclude dead cells. GFP-low and GFP-high fractions were defined from AID-Usp9x cells as indicated. Corresponds to Figs. 1, 3 and 4a, and Supplementary Figs. 1, 2, 4 and 5a. **e)** Sample sort of ES cells carrying the FUCCI cell cycle reporter[5]. mCherry+ and BFP+ populations correspond to the indicated fractions of the cell cycle. Corresponds to Supplementary Fig. 1j.

SUPPLEMENTARY REFERENCES

1. Kalkan, T. *et al.* Tracking the embryonic stem cell transition from ground state pluripotency. *Development* **144**, 1221–1234 (2017).

2. Bulut-Karslioglu, A. *et al.* Inhibition of mTOR induces a paused pluripotent state. *Nature* **540**, 119–123 (2016).

3. Gao, Y. *et al.* Protein Expression Landscape of Mouse Embryos during Pre-implantation Development. *Cell Reports* **21**, 3957–3969 (2017).

4. Israel, S. *et al.* An integrated genome-wide multi-omics analysis of gene expression dynamics in the preimplantation mouse embryo. *Sci Rep-uk* **9**, 13356 (2019).

5. Nora, E. P. *et al.* Targeted Degradation of CTCF Decouples Local Insulation of Chromosome Domains from Genomic Compartmentalization. *Cell* **169**, 930-933.e22 (2017).

6. Blaschke, K. *et al.* Vitamin C induces Tet-dependent DNA demethylation and a blastocyst-like state in ES cells. *Nature* **500**, 222–226 (2013).

7. DiTroia, S. P. *et al.* Maternal vitamin C regulates reprogramming of DNA methylation and germline development. *Nature* **573**, 271–275 (2019).

8. Kuleshov, M. V. *et al.* Enrichr: a comprehensive gene set enrichment analysis web server 2016 update. *Nucleic Acids Res* **44**, W90–W97 (2016).

9. Pasini, D. *et al.* JARID2 regulates binding of the Polycomb repressive complex 2 to target genes in ES cells. *Nature* **464**, 306–310 (2010).

10. Ku, M. *et al.* Genomewide Analysis of PRC1 and PRC2 Occupancy Identifies Two Classes of Bivalent Domains. *Plos Genet* **4**, e1000242 (2008).

11. Yang, P. *et al.* Multi-omic Profiling Reveals Dynamics of the Phased Progression of Pluripotency. *Cell Syst* **8**, 427-445.e10 (2019).

12. Beccari, L. *et al.* Multi-axial self-organization properties of mouse embryonic stem cells into gastruloids. *Nature* 1–27 (2018) doi:10.1038/s41586-018-0578-0.

13. Wang, C. *et al.* Reprogramming of H3K9me3-dependent heterochromatin during mammalian embryo development. *Nature Cell Biology* 1–23 (2018) doi:10.1038/s41556-018-0093-4.

14. Marks, H. *et al.* The Transcriptional and Epigenomic Foundations of Ground State Pluripotency. *Cell* **149**, 590–604 (2012).

15. Morey, L., Aloia, L., Cozzuto, L., Benitah, S. A. & Di Croce, L. RYBP and Cbx7 Define Specific Biological Functions of Polycomb Complexes in Mouse Embryonic Stem Cells. *Cell Reports* **3**, 60–69 (2013).
