# Supplementary material for: The deubiquitinase Usp9x regulates PRC2-mediated chromatin reprogramming during mouse development
Source: Nat Commun. 2021 Mar 25;12:1865. doi: 10.1038/s41467-021-21910-0 (PMC7994559; doi:10.1038/s41467-021-21910-0)
Supplement: Supplementary file 3 — Description of Additional Supplementary Files [file 41467_2021_21910_MOESM3_ESM.pdf]

Description of Additional Supplementary Files

Supplementary Data 1: RNA-seq results from ES cells. Toptable analyses of Usp9x-high and Usp9x-low ES cells after 8h auxin treatment or following 48h washout in serum/LIF. See Fig. 1 and Supplementary Figs. 1-2.

Supplementary Data 2: RNA-seq results from E8.5 embryos. Toptable analyses of Usp9x-mutant versus control embryos dissected at E8.5. Significant genes are defined as padj < 0.1. See Fig. 2 and Supplementary Fig. 3.

Supplementary Data 3: Oligonucleotide sequences. Primer sequences used for cloning, qRT-PCR and mouse genotyping.
