# Supplementary material for: The deubiquitinase Usp9x regulates PRC2-mediated chromatin reprogramming during mouse development
Source: Nat Commun. 2021 Mar 25;12:1865. doi: 10.1038/s41467-021-21910-0 (PMC7994559; doi:10.1038/s41467-021-21910-0)
Supplement: Supplementary file 7 — Reporting Summary [file 41467_2021_21910_MOESM7_ESM.pdf]

nature research

Corresponding author(s): Miguel Ramalho-Santos

Last updated by author(s): Jan 9, 2021

Reporting Summary

Nature Research wishes to improve the reproducibility of the work that we publish. This form provides structure for consistency and transparency in reporting. For further information on Nature Research policies, see our Editorial Policies and the Editorial Policy Checklist.

Statistics

For all statistical analyses, confirm that the following items are present in the figure legend, table legend, main text, or Methods section.

n/a	Confirmed	
☐	☒	The exact sample size (n) for each experimental group/condition, given as a discrete number and unit of measurement
☐	☒	A statement on whether measurements were taken from distinct samples or whether the same sample was measured repeatedly
☐	☒	The statistical test(s) used AND whether they are one- or two-sided<br>*Only common tests should be described solely by name; describe more complex techniques in the Methods section.*
☐	☒	A description of all covariates tested
☐	☒	A description of any assumptions or corrections, such as tests of normality and adjustment for multiple comparisons
☐	☒	A full description of the statistical parameters including central tendency (e.g. means) or other basic estimates (e.g. regression coefficient) AND variation (e.g. standard deviation) or associated estimates of uncertainty (e.g. confidence intervals)
☐	☒	For null hypothesis testing, the test statistic (e.g. F, t, r) with confidence intervals, effect sizes, degrees of freedom and P value noted<br>*Give P values as exact values whenever suitable.*
☒	☐	For Bayesian analysis, information on the choice of priors and Markov chain Monte Carlo settings
☒	☐	For hierarchical and complex designs, identification of the appropriate level for tests and full reporting of outcomes
☒	☐	Estimates of effect sizes (e.g. Cohen's d, Pearson's r), indicating how they were calculated

Our web collection on statistics for biologists contains articles on many of the points above.

Software and code

Policy information about availability of computer code

Data collection	Data were sequenced on Illumina HiSeq 4000 and NextSeq 500 machines.
Data analysis	RNA-seq/ChIP-seq:<br>read trimming and quality checking - Trim Galore! (v0.4.0)<br>genome alignment - TopHat2 (v2.0.13), bowtie2 (v2.2.5)<br>bam file processing - samtools (v1.9), picard (v2.18.14), deepTools (v3.3.1), bedtools v2.28.0<br>read counting - featureCounts, subread v1.5.0<br>data analysis - R Studio (v3.6.1), DESeq2 (v2.0.13), edgeR (v3.26.8), ggplot2 (v3.3.2), tidyverse (v1.2.1)<br>Source code is available on Github: https://github.com/tmacster/usp9x-project-scripts<br><br>ontology and enrichment - David 6.8, geneontology.org, Enrichr (https://amp.pharm.mssm.edu/Enrichr/), GSEAPreranked v6.0.12 (https://cloud.genepattern.org/gp/pages/login.jsf).<br><br>GraphPad Prism v8<br>FlowJo v10.4.2<br>Fiji v2.0.0

For manuscripts utilizing custom algorithms or software that are central to the research but not yet described in published literature, software must be made available to editors and reviewers. We strongly encourage code deposition in a community repository (e.g. GitHub). See the Nature Research guidelines for submitting code & software for further information.

Data

Policy information about availability of data

All manuscripts must include a data availability statement. This statement should provide the following information, where applicable:

- Accession codes, unique identifiers, or web links for publicly available datasets
- A list of figures that have associated raw data
- A description of any restrictions on data availability

RNA-seq and ChIP-seq data generated in this study have been deposited in Gene Expression Omnibus (GEO) under accession number GSE146800 ([https://www.ncbi.nlm.nih.gov/geo/query/acc.cgi?acc=GSE146800]). Published datasets are available from Li et al. (GSE97805 [https://www.ncbi.nlm.nih.gov/geo/query/acc.cgi?acc= GSE97805]), Beccari et al. (GSE113885, [https://www.ncbi.nlm.nih.gov/geo/query/acc.cgi?acc= GSE113885])), van Mierlo et al. (GSE101675, [https://www.ncbi.nlm.nih.gov/geo/query/acc.cgi?acc= GSE101675]), Wang et al. (GSE97778, [https://www.ncbi.nlm.nih.gov/geo/query/acc.cgi?acc= GSE97778]), Liu et al. (GSE73952, [https://www.ncbi.nlm.nih.gov/geo/query/acc.cgi?acc= GSE73952]) and Morey et al. (GEO GSE42466, [https://www.ncbi.nlm.nih.gov/geo/query/acc.cgi?acc=GSE42466]). Source data are provided with this paper.

Field-specific reporting

Please select the one below that is the best fit for your research. If you are not sure, read the appropriate sections before making your selection.

☒ Life sciences ☐ Behavioural & social sciences ☐ Ecological, evolutionary & environmental sciences

For a reference copy of the document with all sections, see nature.com/documents/nr-reporting-summary-flat.pdf

Life sciences study design

All studies must disclose on these points even when the disclosure is negative.

Sample size	Sample sizes were chosen based on reproducibility standards of prior published studies. Specifically, 3 biological replicates were performed for RNA-seq experiments in ES cells. 2 biological replicates were performed per sample for ChIP-seq. At least 2 replicates were performed per condition for western blots. For embryo RNA-seq, 3 replicates of mutants and controls were collected from 2 separate litters for a total of 6 biological replicates. Replicate number was selected for RNA-seq statistical power (≥n = 3) and to ensure biological replication between separate litters. Litter-matched control/mutant pairs were used to minimize biological variability.
Data exclusions	1 biological replicate was excluded from the RNA-seq analysis (8h Usp9x-high ES cells) due to insufficient sequencing depth for quantitative analysis.
Replication	All experiments were replicated a minimum of 2 times, with 3 replicates for RNA-seq experiments and other quantitative analyses. All attempts at reproduction were successful within the reported level of variation. For biochemistry experiments with 2 replicates, orthogonal experiments with independent cell lines were used to test reproducibility of results.
Randomization	For in vitro experiments, control (no-auxin) and treatment (+ auxin) were derived from the same plate of cells to minimize deviation in tissue culture prior to the experiment. No randomization was required for design of in vivo experiments, where mice differed by genotype (Cre+/Usp9x-mutant vs Cre-/Usp9x-fl) rather than treatment or exposure.
Blinding	Investigators were not blinded to the identity of samples to ensure appropriate data collection and because experiments are quantitative in nature, not readily subject to investigator bias. To ensure consistent experimental conditions, all control and treatment samples were processed in parallel.

Reporting for specific materials, systems and methods

We require information from authors about some types of materials, experimental systems and methods used in many studies. Here, indicate whether each material, system or method listed is relevant to your study. If you are not sure if a list item applies to your research, read the appropriate section before selecting a response.

Materials & experimental systems

n/a	Involved in the study	
☐	☒	Antibodies
☐	☒	Eukaryotic cell lines
☒	☐	Palaeontology and archaeology
☐	☒	Animals and other organisms
☒	☐	Human research participants
☒	☐	Clinical data
☒	☐	Dual use research of concern

Methods

n/a	Involved in the study	
☐	☒	ChIP-seq
☐	☒	Flow cytometry
☒	☐	MRI-based neuroimaging

Antibodies

Antibodies used	rabbit anti-Usp9x (Bethyl Laboratories, A301-351A) - 1:2000 for WB, 1 μg for co-IP<br>rabbit anti-H3K27me3 (CST #9733) - 1:1000 for WB, 2.5 μg for ChIP-seq<br>rabbit anti-Suz12 (clone D39F6, C ST #3737) - 1:1000 for WB, 1:50 for IP<br>rabbit anti-Ezh2 ((D2C9) XP CST #5246) - 1:2000 for WB, 1:300 for IP<br>rabbit anti-β-actin (Abcam ab8227) - 1:1000 for WB<br>rabbit anti-H3 (Abcam ab1791) - <1:3000 for WB<br>rabbit anti-Nanog (CST #4903) - 1:1000 for WB<br>rabbit anti-Oct4 (Santa Cruz SC-9081) - 1:1000 for WB<br>mouse anti-HA (Abcam ab9110) - 2.5 μg for HA-Ub IP<br>rabbit IgG (Millipore CS200581) - 1:50 for IP<br>mouse anti-Flag (Sigma F1804) - 1:1000 for WB<br>mouse anti-Gapdh (Millipore MAB-374) - 1:2000 for WB<br>Flag M2-bound magnetic agarose beads (Sigma)<br>GFP-Trap beads (ChromoTek)
Validation	All antibodies used are commercially available and were used according to manufacturer's instructions. Information about usage, validation, and citations are accessible at the product pages:<br>Usp9x: https://www.bethyl.com/product/A301-351A and response to AID knockdown (Fig. 1a, Supplementary Fig. 1a,b).<br>H3K27me3: https://www.cellsignal.com/products/primary-antibodies/tri-methyl-histone-h3-lys27-c36b11-rabbit-mab/9733<br>Suz12: https://www.cellsignal.com/products/primary-antibodies/suz12-d39f6-xp-rabbit-mab/3737<br>Ezh2: https://www.cellsignal.com/products/primary-antibodies/ezh2-d2c9-xp-rabbit-mab/5246<br>β-actin: https://www.abcam.com/beta-actin-antibody-ab8227.html<br>H3: https://www.abcam.com/histone-h3-antibody-nuclear-loading-control-and-chip-grade-ab1791.html<br>Nanog: https://www.cellsignal.com/products/primary-antibodies/nanog-d73g4-xp-rabbit-mab/4903<br>Oct4: http://datasheets.scbt.com/sc-9081.pdf<br>HA: https://www.abcam.com/ha-tag-antibody-chip-grade-ab9110.html<br>IgG: https://www.emdmillipore.com/US/en/product/Normal-Rabbit-IgG,MM_NF-12-370<br>Flag M2: https://www.sigmaaldrich.com/catalog/product/sigma/f1804?lang=en&region=US<br>Gapdh: https://www.emdmillipore.com/US/en/product/Anti-Glyceraldehyde-3-Phosphate-Dehydrogenase-Antibody-clone-6C5,MM_NF-MAB374<br>GFP-trap: https://www.chromotek.com/products/detail/product-detail/gfp-trap/

Eukaryotic cell lines

Policy information about cell lines

Cell line source(s)	Low-passage OsTir1 ES cells were a gift of Elphege Nora and Benoit Bruneau and were used to generate Usp9x-targeted cell lines. HEK293T cells were obtained from ATCC.
Authentication	Cell lines were validated by PCR, immunofluorescence, immunoprecipitation for GFP and/or Flag tags, and flow cytometry for GFP response to auxin.
Mycoplasma contamination	Cell lines tested negative for mycoplasma contamination by PCR-based testing.
Commonly misidentified lines<br>(See ICLAC register)	No commonly misidentified cell lines were used in this study.

Animals and other organisms

Policy information about studies involving animals; ARRIVE guidelines recommended for reporting animal research

Laboratory animals	Usp9xfl/fl females were maintained as homozygotes on a C57BL/6 background by crossing Usp9xfl/fl and Usp9xfl/Y mice20. Heterozygous male Sox2-Cre mice were obtained from Jackson Laboratories (JAX stock #008454) and bred with Cre-negative females to maintain a stock of heterozygous males 63. All mice were housed at 65-75˚ with 40-60% humidity, maintained on a 12h light/dark cycle and provided with food and water ad libitum in individually ventilated units (Techniplast) in specific pathogen-free facilities at The Center for Phenogenomics, Toronto.
Wild animals	This study did not involve wild animals.
Field-collected samples	This study did not involved any samples collected in the field.
Ethics oversight	Experiments were performed according to the Animals for Research Act of Ontario and the Guidelines of the Canadian Council on Animal Care. Animal Care Committee reviewed and approved all procedures conducted on animals at The Center for Phenogenomics, Toronto (Protocol 22-0331).

Note that full information on the approval of the study protocol must also be provided in the manuscript.

ChIP-seq

Data deposition

☒ Confirm that both raw and final processed data have been deposited in a public database such as GEO.

☒ Confirm that you have deposited or provided access to graph files (e.g. BED files) for the called peaks.

Data access links<br>*May remain private before publication.*	Data are deposited in Gene Expression Omnibus (GEO) under accession number GSE146800.
Files in database submission	processed: neg_K27_rep1.bw, neg_K27_rep2.bw, usp9x_low_K27_rep1.bw, usp9x_low_K27_rep2.bw, usp9x_high_K27_rep1.bw, usp9x_high_K27_rep2.bw<br>raw: input_K27_rep1.fq.gz, input_K27_rep2.fq.gz, neg_K27_rep1.fq.gz, neg_K27_rep2.fq.gz, usp9x_low_K27_rep1.fq.gz, usp9x_low_K27_rep2.fq.gz, usp9x_high_K27_rep1.fq.gz, usp9x_high_K27_rep2.fq.gz
Genome browser session<br>(e.g. UCSC)	UCSC genome browser link with replicate-merged bigwigs for 8h Usp9x-low/Usp9x-high (see Supplementary Fig. 4d) as well as individual replicate bigwigs: https://genome.ucsc.edu/s/tmac/tm%2DH3K27me3%2Dbw.

Methodology

Replicates	2 biological replicates were performed per condition. Correlation between replicates was checked by multiBigWigSummary bins and plotCorrelation, and scaled bw files were merged (bigwigCompare add) for heatmaps or analyzed independently.
Sequencing depth	SE reads of 50bp length, with sequencing depth ranging between 23 million-39 million reads per sample (~70% unique mapping rate per sample).
Antibodies	H3K27me3 (CST #9733)
Peak calling parameters	Deduplicated bam files were converted to scaled bedgraphs using deepTools bamCoverage (options --scaleFactor <NF> --binSize 10 --blackListFileName ENCODE_mm10_blacklist.bed) and then to bed files: awk '{print $1"\t"$2"\t"$3"\tid-"NR"\t"$4"\t."}'. These scaled bed files were used to call broad peaks compared to input using epic2 on the command line (options -gn mm10, -d chrM). Bedtools merge was used to merge peaks within 3kb, and bedtools intersect was used to determine a set of common peaks between replicates.
Data quality	Reads were checked for quality and trimmed of adapters using Trim_Galore! (Babraham) according to default recommended parameters, aligned to mm10 or hg19, and duplicated reads removed with Picard MarkDuplicates.
Software	trim_galore 0.4.0, Bowtie2 v 2.2.5, picard MarkDuplicates 2.18.14, R version 3.6.1, deepTools 3.3.1

Flow Cytometry

Plots

Confirm that:

☒ The axis labels state the marker and fluorochrome used (e.g. CD4-FITC).

☒ The axis scales are clearly visible. Include numbers along axes only for bottom left plot of group (a 'group' is an analysis of identical markers).

☒ All plots are contour plots with outliers or pseudocolor plots.

☒ A numerical value for number of cells or percentage (with statistics) is provided.

Methodology

Sample preparation	ES cells were trypsinized, washed, and resuspended in FACS buffer (PBS with 10% FBS) with SYTOX Blue for live/dead analysis.
Instrument	FACS AriaII (BD Biosciences), Sony SH800 Single Cell Sorter (Sony)
Software	FlowJo v10.4.2, Prism v7-8
Cell population abundance	Usp9x-low and Usp9x-high cells correspond to the bottom and top ~15-20% of GFP-AID-Usp9x ES cells, respectively. Purity was confirmed during flow cytometry setup and post-sort.
Gating strategy	Samples were gated by FSC-A/SSC-A, then FSC-W/FSC-A and SSC-A/SSC-W. In pilot experiments, wild-type ES cells were used to determine the range of GFP-negative expression for sorting Usp9x-low ES cells upon auxin treatment (bottom ~15-20% of population), which was then applied to subsequent experiments. Gates for Usp9x-high (GFP-high) ES cells were set to collect a comparable fraction of the total population. Sytox Blue staining (Sytox-negative) was used to isolate intact/live cells.

☒ Tick this box to confirm that a figure exemplifying the gating strategy is provided in the Supplementary Information.
